# Linkage Disequilibrium in Wild Mice

**DOI:** 10.1371/journal.pgen.0030144

**Published:** 2007-08-24

**Authors:** Cathy C Laurie, Deborah A Nickerson, Amy D Anderson, Bruce S Weir, Robert J Livingston, Matthew D Dean, Kimberly L Smith, Eric E Schadt, Michael W Nachman

**Affiliations:** 1 Department of Biostatistics, University of Washington, Seattle, Washington, United States of America; 2 Rosetta Inpharmatics, Seattle, Washington, United States of America; 3 Department of Genome Sciences, University of Washington, Seattle, Washington, United States of America; 4 Department of Ecology and Evolution, University of Arizona, Tucson, Arizona, United States of America; Stanford University School of Medicine, United States of America

## Abstract

Crosses between laboratory strains of mice provide a powerful way of detecting quantitative trait loci for complex traits related to human disease. Hundreds of these loci have been detected, but only a small number of the underlying causative genes have been identified. The main difficulty is the extensive linkage disequilibrium (LD) in intercross progeny and the slow process of fine-scale mapping by traditional methods. Recently, new approaches have been introduced, such as association studies with inbred lines and multigenerational crosses. These approaches are very useful for interval reduction, but generally do not provide single-gene resolution because of strong LD extending over one to several megabases. Here, we investigate the genetic structure of a natural population of mice in Arizona to determine its suitability for fine-scale LD mapping and association studies. There are three main findings: (1) Arizona mice have a high level of genetic variation, which includes a large fraction of the sequence variation present in classical strains of laboratory mice; (2) they show clear evidence of local inbreeding but appear to lack stable population structure across the study area; and (3) LD decays with distance at a rate similar to human populations, which is considerably more rapid than in laboratory populations of mice. Strong associations in Arizona mice are limited primarily to markers less than 100 kb apart, which provides the possibility of fine-scale association mapping at the level of one or a few genes. Although other considerations, such as sample size requirements and marker discovery, are serious issues in the implementation of association studies, the genetic variation and LD results indicate that wild mice could provide a useful tool for identifying genes that cause variation in complex traits.

## Author Summary

Linkage disequilibrium (LD) refers to the nonrandom association of variants at different sites in the genome. In recent years, LD has been of great interest in biomedical research because of its utility in “association studies,” where DNA sequence variants associated with disease traits are used to identify susceptibility genes. The resolution of this gene-finding tool depends on how the LD decays with distance between the associated sites. The pattern of LD decay is well known in human populations, where it provides high resolution on the order of one or a few genes. This paper shows that the pattern of LD in wild house mice (in contrast to laboratory mice) is very similar to that in human populations. This result means that wild mice (reared in the laboratory) could be used in association studies to identify genes that cause trait variation. Wild mouse association studies might complement those in humans by dealing with traits that are difficult to measure in humans (such as response to carcinogen exposure) and by filtering human associations for subsequent validation with genetically engineered mouse models.

## Introduction

The house mouse, *Mus musculus,* consists of three principal subspecies, with native populations of *M. m. musculus* in Eastern Europe and Asia, *M. m. castaneus* in Southeast Asia and India, and *M. m. domesticus* in Western Europe and the Middle East [1]. Populations in the Americas, Africa, and Australia are mainly of *domesticus* origin, due to recent transportation by Western European seafarers [1], although an admixture of *domesticus* and *castaneus* has been found in one California population [2], and other such cases may occur elsewhere. M. musculus developed a commensal relationship with humans at the dawn of agriculture, and natural populations (“wild” mice) of all three subspecies now live primarily in close association with human dwellings [3]. Laboratory strains were developed from domesticated pets and appear to be an admixture of all three subspecies [4,5].

Crosses between inbred strains of laboratory mice have been used very successfully to identify quantitative trait loci (QTL) that affect a variety of complex traits, including many related to human diseases such as atherosclerosis, diabetes, and obesity. Flint et al. [6] note that more than 2,000 mouse QTL have been detected, whereas only about 20 of the causal genes have been identified. The basic problem in gene identification is that most QTL have been discovered in F2 mapping populations, in which the confidence region for a QTL generally spans 20–40 cM (containing hundreds of genes), and efficient methods for fine-mapping have not been available. The classical approach to fine-mapping is construction of congenic lines, which can take several years to achieve a resolution of 1 cM (containing about 15 genes) [6].

In recent years, new genetic and genomic approaches have been used to reduce the time and improve the resolution of QTL mapping in mice (Table 1). These approaches include haplotype analysis of parental lines to exclude regions of identity-by-descent [7,8], association studies with a set of inbred lines [9–11], and admixture mapping in laboratory stocks with multiple inbred parents and multiple generations of recombination [12]. The expected resolution of several methods is an interval containing roughly ten to 20 genes, although the actual resolution varies with other factors such as gene density. The use of transcriptional profiling and other functional annotation sometimes can reduce the number of likely candidates to just one or a few genes [12–15]. However, useful annotations are not always available, and could be misleading. Hence, there is a need for an efficient genetic method that achieves even finer mapping resolution than existing methods.

**Table 1:**
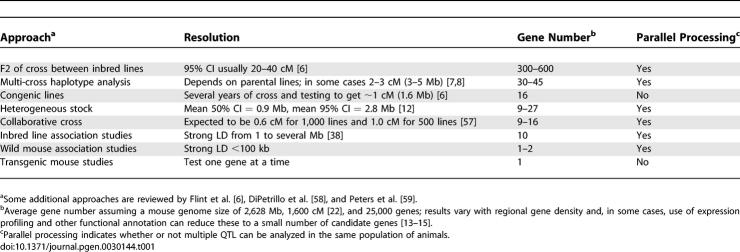
Genetic Approaches to Identification of QTL in Mice

In humans, association studies are being used extensively to identify genes that cause variation in complex traits [16–18]. This method relies on either genotyping the causative polymorphism directly, or on genotyping “tag” markers that are in strong linkage disequilibrium (LD) with the causative site. In human populations, strong LD occurs over a distance that varies depending on the genomic region, but is usually on the order of tens of kilobases [19–21]. Therefore, a significant marker–trait association in humans usually indicates that a causative polymorphism for the trait is located within about 100 kb of the marker, providing a resolution on the order of one or a few genes for LD mapping. This pattern of LD has been shaped by recombination, population structure, and demographic history. Although the house mouse has less recombination than humans [22], the commensal relationship between the two species has resulted in parallels in demographic history that might have led to similarities in the LD pattern.

Here, we investigate the genetic structure of a natural population of mice in Arizona to determine its suitability for fine-scale LD mapping and association studies. There are three main findings: (1) Arizona mice have a high level of genetic variation, which includes a large fraction of the sequence variation present in classical strains of laboratory mice; (2) they show clear evidence of local inbreeding but appear to lack stable population structure across the study area; and (3) LD decays with distance at a rate similar to human populations, which is considerably more rapid than in laboratory populations of mice. These results indicate that wild mice could provide a useful tool for identifying genes that cause variation in complex traits.

## Results/Discussion

### Genetic Variation in Wild Mice

In this study, wild mice *(M. musculus domesticus)* were sampled from a natural population in the vicinity of Tucson, Arizona. A total of 94 mice were collected, each from a different site to avoid sampling close relatives, and we also collected small samples of the three M. musculus subspecies from their native ranges. To assess population structure and long-range LD, these mice were genotyped for a genome-wide set of 4,581 single nucleotide polymorphisms (SNPs) with a median distance between adjacent markers of ∼500 kb. These SNPs were ascertained in laboratory strains. In the Arizona mice, 89% of the SNPs are polymorphic, and 67% are “common” (i.e., minor allele frequency [MAF] > 0.05).

To assess short-range LD, we resequenced segments in four genomic regions, each on a different autosome, and each focused on a candidate gene from previous QTL studies of metabolic traits in laboratory mice (*Alox15* [23], *Apoa2* [13], *C3ar1* [24], and *Nr1h3* [25]). A total of 25.7 kb of sequence per mouse was obtained in segments of ∼1–2 kb, with one segment in each gene of interest and the others at distances of 25, 100, and 200 kb away from the gene in a single direction. The observed nucleotide diversity (π, the average proportion of nucleotide differences between pairs of sequences) is 0.10% in *Alox15,* 0.37% in *Apoa2,* 0.45% in *C3ar1,* and 0.09% in *Nr1h3* (excluding coding sequence). Although the sample of loci sequenced in the Arizona mice is small, the mean π (0.25%) is very similar to the mean of 0.21% (SD = 0.20) for 21 autosomal loci in Western European populations of *domesticus* (the source of North American populations) [26,27]. This result indicates a high level of genetic variation in Arizona mice. For reference, human populations of Asian and European descent have an estimated π of 0.08% to 0.10% for autosomal loci [28,29].

The wild Arizona mice are polymorphic for a large fraction of SNPs found in classical inbred lines of laboratory mice. A total of 285 SNPs were discovered by resequencing the four regions in Arizona mice, and 163 of these are common (MAF > 0.05). In the same segments, public databases contain 63 SNPs that are polymorphic across classical inbred lines. The public databases do not contain a complete inventory of all SNPs that occur in laboratory strains, so these SNPs should be regarded as a sample. Within the sample of 63 classical line SNPs, 51% (32) occur with MAF > 0.05 in Arizona mice. Similarly, 67% of the 4,581 SNPs in the genome-wide panel (ascertained in laboratory strains) are common in the Arizona population. These results suggest that roughly 50% of the QTL found in classical line crosses will be segregating in the Arizona population, assuming that most QTL are due to SNPs (rather than structural variation [30]) and that causal SNPs are distributed in the same way as other SNPs.

In a related study, T. Salcedo and M. Nachman (unpublished data) examined haplotypes of five X-linked loci in European *domesticus* and *musculus* subspecies, as well as eight classical inbred strains. They found that laboratory strain haplotypes often are common in the wild mouse populations, consistent with earlier studies comparing wild-derived and classical inbred lines [4]. These haplotype studies support the notion that many of the lab mouse QTL are common in wild mouse populations. In addition, because laboratory mice contain a limited sample of the genetic diversity that occurs in nature [5], wild mice also provide an opportunity for new QTL discovery.

### Population Structure

The Arizona mice show significant deviations from the Hardy-Weinberg (HW) equilibrium, with an excess of homozygotes at 45% of autosomal loci (α = 0.05) in the genome-wide panel. The inbreeding coefficient estimated from HW deviations is 0.20 averaged across loci. A similar study by Ihle et al. [31] of 204 microsatellite loci in *domesticus* populations in France, Germany, and Africa also revealed excess homozygosity. These results suggest the presence of population structure and/or inbreeding in wild mouse populations, as previously indicated by allozyme and other field studies of *domesticus* populations [32,33].

**Figure 1:**
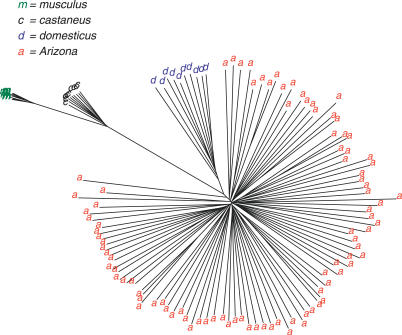
A Neighbor-Joining Tree of Wild-Caught House Mice Based on Allele-Sharing at 4158 SNP Loci The individuals include ten *M. m. domesticus* from Western Europe, seven *M. m. musculus* from Eastern Europe, nine *M. m. castaneus* from India, and 94 mice from Tucson, Arizona. The Arizona mice appear closely related to *domesticus* from Western Europe, as expected from the history of North American colonization and other evidence [1]. The small terminal branch lengths of *musculus* and *castaneus* may be due to an ascertainment bias in the SNPs, which were discovered in laboratory strains in which the nuclear genome seems to be predominantly of *domesticus* origin [4,56].

The relationships among wild mice were investigated by clustering genetic distances (one minus the mean fraction of alleles shared per locus). Figure 1 shows a neighbor-joining tree for samples from Arizona and for the three subspecies from their native ranges. The star-shaped pattern for Arizona mice indicates a lack of geographic differentiation within the study area and suggests that most pairs are unrelated. Very similar trees were obtained by Ihle et al. [31] for samples from French, German, and African populations of *domesticus.* There is no overall correlation between genetic distance and collection site distance in Arizona (Figure S1), although several pairs of individuals with low genetic distances also have low collection site distances. This result indicates some local inbreeding, but argues against a gradient of differentiation across the study area.

We also looked for cryptic structure in the Arizona population using the model-based clustering method of Pritchard et al. as implemented in the Structure 2.2 program [34]. When all the individuals in Figure 1 are used in the analysis, there is a large increase in the likelihood going from a model of one to a model of two subpopulations, but very small changes going from two to three or from three to four subpopulations (Figure S2A). The two-subpopulation model separates the *musculus, castaneus* pair from the Arizona, western European *domesticus* pair, such that each individual is clearly assigned to one or the other group. The three- and four-subpopulation models also maintain this division. Analysis of the Arizona population alone gives only small changes in the likelihood with each increase in the number of subpopulations modeled (Figure S2B). Therefore, the model-based structure analysis fails to support differentiation of the Arizona population into a small number of subpopulations.

We also estimated the inbreeding coefficient of each individual and the kinship coefficient of each pair of individuals using a model that allows for inbreeding, but no population structure. The mean of the inbreeding coefficients is 0.21 (consistent with the observed deviations from the HW equilibrium) and 90 of the 94 estimates are less than 0.5 (Figure S3). Four mice have inbreeding coefficients greater than 0.50, which is equivalent to three generations of full-sibling mating. The mean kinship coefficient is 0.0055, and 91% of the 4,371 pairs have an estimate less than 0.0156, which is the expected value for second cousins in a population with no inbreeding (Figure S4). Therefore, most pairs of mice are essentially unrelated, as indicated by the neighbor-joining tree in Figure 1, but a small number are rather highly inbred, and several pairs are closely related (see Figures S3 and S4).

Early studies of allozyme variation in North American *domesticus* showed genetic differentiation on a fine spatial scale, such as different buildings on the same farm, suggesting highly structured populations and low dispersal rates [32,35]. However, recent ecological studies have shown that wild mouse demes are very transient in nature, and there is considerable long-distance migration [36]. The results presented here are consistent with the ecological studies in suggesting some local inbreeding (which may result in transient differentiation on a fine scale), but also considerable gene flow across distances on the order of tens of kilometers so that stable population structure within our study area appears to be absent.

### LD

**Figure 2:**
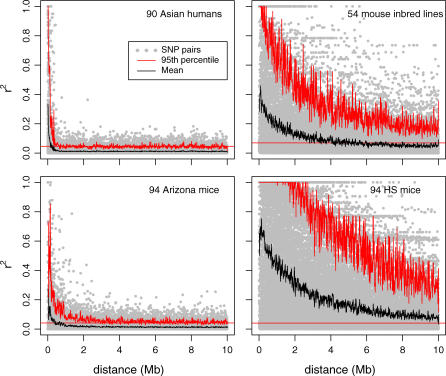
LD between Pairs of ∼2,900 Autosomal SNPs in Humans and Mice The *r* ^2^ variable is the composite (genotypic) measure of LD, which is very similar to the usual gametic measure (see Materials and Methods). The jagged red line is the 95th percentile of the *r* ^2^ values within a 25 kb sliding window. The horizontal red line is the genome-wide threshold for significance of *r* ^2^ at α = 0.05 (calculated as the 95th percentile of permuted data). The jagged black line is the mean of the *r* ^2^ values within a 25 kb sliding window. HS is an outbred laboratory strain.

The decay of LD for autosomal SNPs from the genome-wide set is summarized in Figure 2 (and Figure S5), with comparisons to previous studies of human and laboratory mouse populations. The figures show that LD decays with physical distance in Arizona mice on a scale similar to samples from human populations of Asian and European descent [19], although somewhat less rapidly. In the human samples, there is essentially no significant LD between markers >1 Mb apart, but in Arizona mice this distance is >2 Mb. However, in Arizona mice, when markers are more than 200 kb apart, *r* ^2^ is nearly always less than 0.3, which provides very low power for detecting associations unless the effect is very large [37]. The LD for X-linked SNPs may be slightly higher than for autosomal SNPs. In the range of 0–2 Mb, the mean of *r* ^2^ for X-linked pairs is 0.072, and that for autosomal pairs is 0.021, but the sample size of X-linked pairs is small (82 X-linked versus 7,669 autosomal). Permutation testing shows essentially no significant LD between pairs of markers on different chromosomes in Arizona mice (1.3% are significant at the nominal 1% level). This result is consistent with the apparent lack of population structure (despite the excess homozygosity).

Figure 2 also shows LD in two types of laboratory mice that are being used to refine QTL location. One type is a set of 54 inbred lines used for association studies (also known as “in silico” mapping [9]), and the other is an outbred “heterogeneous stock” (HS) founded from an eight-way cross of inbred lines and used for admixture mapping [12]. In contrast to natural populations of mice and humans, these laboratory mice have strong LD for distances up to several megabases, and *r* ^2^ values of 1 are sometimes observed for very distant (even unlinked) markers, as noted previously [12,14,38]. The smaller sample size of inbred lines does not account for the much higher levels of LD, since comparisons with wild mice and humans of equal sample size show similar differences (Figure S5). These results indicate that wild mice have the potential to deliver much finer mapping resolution than laboratory populations.

**Figure 3:**
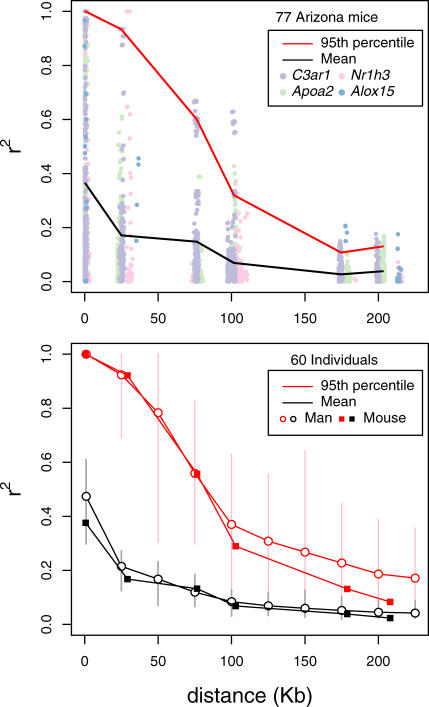
LD between SNPs Discovered by Resequencing in Selected Regions of the Genome, Measured as the Genotypic *r* ^2^ The top panel shows LD in 77 Arizona mice for pairs of SNPs within each of four regions (points) and summary statistics for all four regions combined (lines). The total number is 163 common SNPs (nine *Alox15,* 53 *Apoa2,* 70 *C3ar1,* 31 *Nr1h3*). The lines connect the midpoint of each distance bin and give either the mean or the 95th percentile of *r* ^2^ for that bin. The lower panel compares LD in 60 unrelated individuals each of Arizona mice and humans of European ancestry from the CEU sample of the HapMap project. The mouse SNPs are the same as in the top panel, except that the total number is 141 (removing those with MAF < 0.05 in the sample of 60 individuals). The human SNPs are a subset from 10 ENCODE regions, selected to have the same allelic frequency distribution as the mouse SNPs. The human SNP numbers range from 131 to 803 per region, with a total of 3891 over all ten regions. The ranges of *r* ^2^ statistics (mean or 95th percentile) for the ten ENCODE regions are given by the vertical bars.

The genome-wide set of SNPs assayed in Arizona mice has relatively few pairs at short distances (96 pairs less than 100 kb apart). To assess short-range LD in more detail, we used resequencing data from four genomic regions. Figure 3 shows the pattern of LD decline over 200 kb in 77 Arizona mice for 163 common SNPs. The decrease in *r* ^2^ is similar in all four regions, although somewhat less sharp for *C3ar1.* The 95th percentile of *r* ^2^ falls to less than 0.4 at 100 kb. Since the mouse genome has, on average, about one gene per 100 kb, these results indicate that the pattern of LD in the Arizona population can provide a mapping resolution on the order of one or a few genes.

Figure 3 also compares LD in 60 Arizona mice with 60 unrelated European humans [19]. The human data include 3,891 SNPs selected from ten resequenced regions in order to match the allelic frequency distribution in the Arizona mice. These regions are from the Encyclopedia of DNA Elements (ENCODE) project. The mean *r* ^2^ for SNPs within a 0–2 kb distance is somewhat lower in the Arizona mice (0.38) than in European humans (0.47), but the ranges overlap (0.29 to 0.43 for the four mouse regions and 0.30 to 0.61 for the ten human regions). The pattern of LD decline in these samples is very similar for the two species. This result appears to be somewhat different than the slower decline of LD in mice over longer distances (Figure 2), which may be due to the small sample of genomic regions resequenced in the Arizona mice, since LD is known to vary considerably among regions in humans [19]. In any case, the differences are fairly small at both long- and short-range distances, suggesting that the resolution of association mapping in Arizona mice would be similar to that in human populations of European and Asian descent.

**Figure 4:**
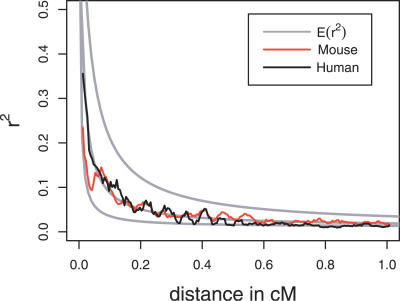
Mean LD in 94 Arizona Mice and 90 Asian Humans, Evaluated in a Sliding Window of 0.025 cM The data are the same as in Figure 2, but physical distance has been converted to genetic distance using genome-wide averages of cM/Mb. The expected value of *r* ^2^ for a sample size of n diploids is calculated as *E* (*r* ^2^) = (1 / (1 + 4*N_e_c*)) + (1 / *n*), where *N_e_* is effective population size and *c* is recombination rate (converted to cM for plotting by the Kosambi mapping function). In this case, *n* = 92 and *N_e_* = 1,000, 3,000, or 10,000 (from top to bottom).

The expected level of LD in a sample at equilibrium under the neutral model depends on the rate of recombination (*c*), the effective population size (*N_e_*), and the diploid sample size (*n*): *E* (*r^2^*) = (1 / (1 + 4*N_e_c*)) + (1 / *n*) [39]. The human genome has an average of about 1.20 cM/Mb (based on a genetic map from families of European descent [40], whereas the mouse has about 0.62 cM/Mb (based on a genetic map from outbred laboratory mouse families [22]). This difference in recombination rate may account, at least in part, for the apparently slower rate of decline in LD over long physical distances in the Arizona mice compared with Asian humans (Figure 2). Figure 4 shows the relationship between LD and genetic distance, in which the latter was calculated from the genome-wide average estimates of cM/Mb. The decline of LD with genetic distance is very similar, except that when the genetic distance is less than 0.05 cM, the mean r^2^ of Arizona mice is less than in Asian humans. Although the sample size for mice in the genome-wide SNP set is small (69 pairs between 0 to 0.05 cM, compared with 230 pairs in Asian humans), the resequencing data in Figure 3 also suggest less LD at this distance. The pattern of LD decline over a short physical distance is very similar for the two species, but the local recombination rates are, on the average, less in the mouse than in humans (0.77 cM/Mb and 0.99 cM/Mb, respectively, in a ∼10 cM window around each region). Nevertheless, because LD varies considerably among regions, firm conclusions about a possible difference in short-range versus long-range LD will require data from additional genomic regions in wild mice.

Although differences in recombination rate appear to account for much of the difference in LD decline in Arizona mice and non-African humans, expected LD is also dependent on population size. Figure 4 shows the expected decline in LD with genetic distance for *N_e_* values of 1,000, 3,000, and 10,000. The expectation for *N_e_* = 3,000 fits the human data very well, but is not consistent with *N_e_* estimates based on nucleotide diversity, which are 8,000–10,000 [29,41]. This observation is well known—i.e., that LD levels in humans of European and Asian descent suggest a smaller effective population size than that indicated by polymorphism levels [41]. The discrepancy appears to be due to departures from demographic equilibrium, and both types of data are compatible with a range of simple bottleneck models for non-African humans [28]. The discrepancy for Arizona mice is even larger, since polymorphism levels are higher (∼0.2% versus ∼0.1%), implying a higher *N_e_*. Wild populations of *domesticus,* like humans, have experienced large range expansion in the past few thousand generations (humans out of Africa and *domesticus* out of the Middle East with agricultural humans [3,42]) and possibly also bottlenecks associated with colonization. Therefore, further work on the comparative population genetics of wild mice and humans may contribute significantly to our understanding of sequence variation and evolution in both species.

### Prospects for Association Studies of Complex Traits in Wild Mice

The results presented here show that the Arizona population of wild mice has a genetic structure that can complement and extend existing methods for identifying quantitative trait genes. It has a high level of genetic variation that captures a large fraction of the polymorphisms (and presumably also the QTL) in laboratory mice. It also has a favorable pattern of LD in that strong associations are limited primarily to markers less than 100 kb apart, which provides the possibility of fine-scale association mapping at the level of one or a few genes.

Favorable patterns of genetic variation and LD are two basic requirements for useful association studies, but there are also important practical considerations such as obtaining an adequate sample size of interesting phenotypes and a sufficiently high density of SNPs to take advantage of the fine-scale LD. These are serious issues, but potentially tractable. Wild mice can be bred easily in the laboratory and phenotyped under controlled conditions, which should reduce the sample size requirements relative to human association studies. Family-based designs could be used to reduce the need to collect large numbers of mice in the wild. Furthermore, transgenic and knockout mouse technologies provide an immediate functional test within the same species for genes with putative associations, thus reducing the need for large replicate cohorts. In the short term, SNPs for association studies could be discovered in candidate genes by standard methods of resequencing. In the long term, new sequencing technologies may make this process much more efficient and less costly.

In recent years, there has been a great deal of investment in whole-genome association studies in humans, and these efforts are coming to fruition [17,18,43,44]. There are two areas in which mouse association studies could complement those in humans. (1) Recent reports of whole-genome association in humans show a small number of hits that are highly reproducible and a much larger number that are promising, but require validation [18,43]. One possibility for validation is transgenic testing in mice, but this process is time consuming and may not be appropriate when homologous genes play different physiological roles in the two species. However, a SNP–trait association for a given gene in both humans and mice would suggest a true positive and also indicate that the trait is sensitive to variation of that gene in both species. Thus, candidate association studies in mice using human whole-genome association hits could provide an indication of whether transgenic testing in mice is likely to provide useful results. (2) Association studies in wild mice could be used for traits that cannot be measured easily in humans, such as response to carcinogen exposure, adverse effects of drugs at high dosage, susceptibility to disease agents, or gene expression in multiple tissues. Furthermore, the ability to control diet and other environmental exposures allows unbiased detection of genotype–environment interactions. Therefore, despite the growing success of human association studies, wild mouse studies have potential for contributing to our understanding of the genetic basis of complex traits of medical importance.

## Materials and Methods

### Sample collection.

All aspects of the study were approved by the Institutional Animal Care and Use Committee of the University of Arizona and were performed in accordance with institutional policy and National Institutes of Health guidelines governing the humane treatment of vertebrate animals. Arizona mice were collected with Sherman traps at 94 different sites (mostly barns and houses) covering an area of 93 × 60 km in and around Tucson. These sites are spaced at least 100 m apart (except for one pair at 60 m), with a median intersite distance of 13.1 km, and only one mouse per site was genotyped. A small sample (seven to ten animals each) of each of the three M. musculus subspecies were trapped from more widely dispersed sites in their native ranges: ten *M. m. domesticus* in Western Europe (Italy, Greece, and Spain), seven *M. m. musculus* in Eastern Europe (Slovakia, Poland, and Hungary), and nine *M. m. castaneus* in India (Katrain, Dehardun, Mandi, and Siliguri). Animals were killed and DNA was extracted from liver using PureGene (Gentra Systems, http://www.qiagen.com). Dataset S1 provides sample annotation.

### Genotyping wild mice.

A genome-wide set of SNPs was genotyped for the 94 Arizona mice and seven to ten each of the subspecies from their native ranges. The genotyping was performed by Affymetrix using their GeneChip Mouse Mapping 5K SNP Kit (http://www.affymetrix.com/support/technical/datasheets/mouse_5k_datasheet.pdf). This chip includes 5,071 assays for SNPs ascertained in laboratory strains of mice, of which 4,581 were successful with the wild mouse samples. The dataset for successful assays is 98% complete over all sites and individuals, and is provided in Datasets S2 and S3. For LD estimates in Figure 2 (94 Arizona mice), we selected 2,974 autosomal SNPs with MAF > 0.05, <10% missing values, and unique genomic location on mouse genome build 36. These markers are well distributed across the genome, with median, mean, and standard deviation of distance between adjacent markers (or marker and chromosome terminus) of 536, 857, and 1,158 kb, respectively. For LD estimates in Figure S5, we selected a subset of 54 Arizona mice at random and 2,859 SNPs with MAF > 0.05 and six or fewer missing values in those individuals.

### Resequencing wild mice.

Sequence was obtained from 77 Arizona mice in each of four genomic regions (a subset of animals genotyped using the Affymetrix chip). Each region consists of four segments of 1–2 kb in length, with one segment in a gene of interest and the others at distances of 25, 100, and 200 kb away from the gene in one direction. Partially overlapping polymerase chain reaction products (two to four per segment) were sequenced using standard Big Dye terminator chemistry and capillary electrophoresis on ABI3730 (Applied Biosystems, http://www.appliedbiosystems.com). Sequence traces were base-called using Phred, assembled into contigs onto the mouse reference sequence, and then scanned for SNPs with Polyphred, version 5.01 [45]. Assembled traces and variant sites were visually inspected using Consed [46] to ensure the accuracy of the alignments and variant calls. This dataset is 90% complete over all sites and individuals and is provided in Datasets S4 and S5. For LD comparison with ENCODE data from 60 humans of European descent, we selected a subset of 60 mice and 141 mouse SNPs with MAF > 0.05 and six or fewer missing genotypes for each SNP in those individuals.

### Public mouse SNPs.

Two public databases, dbSNP mouse build 126 (http://www.ncbi.nlm.nih.gov/projects/SNP) and Perlegen mouse release 3 (http://mouse.perlegen.com/mouse), were searched for mouse SNPs in the genomic regions that were resequenced in the Arizona mice. Overlap between the public and Arizona mouse SNPs was determined by flanking sequence alignment using Cross_Match (http://www.phrap.org). Genotypes were downloaded, and SNPs that occur in classical inbred lines were identified as sites with at least two different genotypes among lines that are not wild derived (i.e., not in the list of wild derived lines provided on the Jackson Laboratory Web site; http://jaxmice.jax.org/list/cat481389.html).

### Statistical analysis.

Genotypic data were analyzed with R statistical software [47]. Exact HW tests were performed with the function “HWE.exact” in the “genetics” package [48]. The within-population inbreeding coefficient, which is the correlation between alleles at one locus within an individual [49], was calculated using the “diseq” function. LD was estimated as the composite (genotypic) *r* ^2^, which is the squared correlation of genotypic indicators at two loci in a diploid individual, whereas the usual gametic *r* ^2^ is the squared correlation of allelic indicators at two loci in a haploid gamete [49]. Since a large fraction of loci show significant deviation from the HW expectation in the Arizona mice, we prefer the genotypic rather than the usual gametic correlation, because its calculation does not require an assumption of random mating. However, to evaluate the potential difference, we used a maximum likelihood method (implemented in the “LD” function of the R genetics package) to estimate the gametic correlation. Although random mating is assumed for this estimation, deviations in the direction of excess homozygosity (as observed in the mice) are not likely to bias the estimate [50]. Figure S6 shows that there is very little difference between the squared genotypic and gametic correlation estimates in either Arizona mouse or human populations. Nevertheless, we present the assumption-free genotypic measure, which may be more relevant in the context of association studies [49]. Permutations (*n* = 1,000) were used to obtain genome-wide significance thresholds for composite *r* ^2^.

### Genetic distance tree.

Genotypes from the genome-wide set of SNPs were used to construct a genetic distance matrix as the mean fraction over loci of the number of shared alleles between each pair of individuals. The loci consist of all SNPs (4,158) that have at least some nonmissing values within each of the four groups of wild-caught mice. This matrix was used to construct a neighbor-joining tree [51] with the “nj” function of the R statistics package “ape” [52].

### Structure analysis.

Structure 2.2 (http://pritch.bsd.uchicago.edu/structure.html) was used to detect cryptic population structure using the model-based approach of Pritchard et al. [34]. This method assumes LD within subpopulations, so the full set of autosomal markers were thinned to a set of 752 in which no two markers were closer than 2 Mb apart (the distance at which there is very little LD). Two sets of individuals were analyzed: (1) the full set of 120 mice shown in Figure 1 and (2) just the 94 Arizona mice. For each set of mice, the admixture model having one, two, three, or four subpopulations was analyzed. For each model and set of mice, three to seven independent runs of the program were made, with burn-in and subsequent steps each numbering 25,000 (or, in some cases, 50,000).

### Relatedness analysis.

Degrees of inbreeding and relatedness in the mice in our study were estimated by maximum likelihood using a model that allows for inbreeding but no population structure, as described in Milligan [53], Hepler [54], and Weir et al. [55]. This model is based on Jacquard's nine identity coefficients, Δ = Δ_1_,…, Δ_9_. Maximum likelihood estimates of the nine coefficients were obtained for each pair of individuals in our sample using an expectation–maximization algorithm. The estimated kinship coefficient, *θ_XY_* for individuals *X* and *Y*, was obtained from the estimates of Δ and the relationship 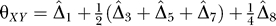 . The inbreeding coefficient for individual *X* (*F_X_*) was estimated as *F_X_* = *2θ_XX_* − *1*, where *θ_XX_* is the kinship coefficient of an individual with itself. The data used for relatedness estimation consist of 3,928 autosomal SNPs from the genome-wide set.

We performed a simulation study in which the performance of the estimators was evaluated with a set of markers similar to those used in this study. Data were simulated via gene-dropping, with 3,928 autosomal markers having allelic frequencies that matched those observed in the Arizona mice. We obtained genetic map positions for 2,087 of the 3,928 markers directly from a map of 8,513 markers [22], and the remaining positions were estimated by interpolation. Recombination frequencies for pairs of adjacent loci were obtained using Haldane's map function. In the simulations of meioses from parent to offspring, crossovers occurred along each chromosome according to a no-interference model. We simulated individuals and pairs of individuals with a variety of different levels of relatedness and inbreeding. In general, estimated kinship coefficients were within 0.063 units of the true values in 95% of all simulated pairs of relatives. Estimated inbreeding coefficients were within 0.120 units of the true values in 95% of all simulated individuals. Estimation was more accurate for certain situations: the estimated inbreeding coefficient was below 0.031 for 95% of all simulated non-inbred individuals, and the estimated kinship coefficient was below 0.015 for 95% of all simulated pairs of non-inbred unrelated individuals.

### Genome-wide LD in mouse inbred lines.

We started with the 60 classical and wild-derived inbred lines of Petkov et al. [38], which were selected for their genetic diversity and to remove closely related “sibling” strains. Six of these lines were eliminated for lack of sufficient genotypic data (LT/SvEiJ, CAST/EiJ, MOLD/RkJ, CZECH/EiJ, SKIVE/EiJ, and PWK/PhJ). Genotypes for the remaining 54 lines were obtained from the Wellcome-CTC Mouse Strain SNP Genotype Set Web site (http://www.well.ox.ac.uk/mouse/INBREDS). From a total of 12,614 SNPs that are polymorphic across the 54 lines, we selected 2,855 autosomal SNPs with six or fewer missing values to match the allelic frequency distribution (within 5% bins) in a random subset of 54 Arizona mice, while maximizing overlap with the Arizona SNPs.

### Genome-wide LD in HS mice.

These data are from the HS QTL project [12], and genotypes were downloaded from their Web site (http://gscan.well.ox.ac.uk). For the LD estimation, we selected either 94 (Figure 2) or 54 (Figure S5) animals at random from a pool of 1,940 animals with identifiers beginning with “A0” (i.e., not ancestors of other animals in the set). These 1,940 animals belong to 85 different families. The samples of 94 or 54 animals belong to 33 or 22 families, respectively. We also analyzed a sample of 85 animals, consisting of one randomly chosen individual per family, and the LD results are very similar. For example, at an intermarker distance of 2 Mb, the mean *r* ^2^ = 0.31 and 0.26 and the 95th percentile of *r* ^2^ is 1.00 and 0.96 for the samples of 85 and 94, respectively. From a total of 12,112 SNPs genotyped in the HS animals, we selected a subset to match the corresponding Arizona set in terms of number and allelic frequency distribution (within 5% bins) while maximizing overlap with the Arizona SNPs. All selected SNPs had <10% missing values.

### Genome-wide LD in humans.

To estimate long-distance LD for a set of SNPs comparable to the Arizona mouse data, we analyzed a selected set of genotypic data from the International HapMap Project [19] (release 21; http://www.hapmap.org). Data for three population samples were used: 60 unrelated individuals with European ancestry (Centre d'Etude du Polymorphisme Humain samples of Utah residents with ancestry from northern and western Europe [CEU]), 45 Japanese from Tokyo, Japan, and 45 Han Chinese from Beijing, China. The two Asian samples were combined for allelic frequency and LD estimation. A SNP set was selected separately for each human group (Asian and European) to match the Arizona mouse set in terms of number, allelic frequency distribution (within 5% bins), and chromosomal distribution (20 linkage groups, excluding human Chromosomes 20–22), but otherwise at random. The two Asian samples were combined for LD estimation by first calculating the genotypic correlations for the Japanese and Chinese samples separately, testing for homogeneity, and then averaging the two correlations using Fisher's *z*-transformation. The two sets of correlations are very homogenous, since 1.1% of the homogeneity tests are significant at the nominal level of 1%. For comparison to the sets of 54 mouse samples, 54 of the 60 CEU samples were chosen at random.

### Short-range LD in humans.

For comparison to LD in SNPs discovered by resequencing Arizona mice, we selected SNPs discovered by resequencing a panel of 16 CEU individuals in 10 ENCODE regions and subsequently genotyped on 90 CEU individuals [19]. For the 60 unrelated CEU individuals, we selected a set of 3,891 SNPs with MAF > 0.05, six or fewer missing values, and an allelic frequency distribution matching the 141 SNPs selected for a set of 60 Arizona mice. Genotypes were obtained from the HapMap Web site.

## Supporting Information

Dataset S1 Wild Mouse Sample Annotation The file contains tab-delimited text in 120 rows and seven columns.
(9 KB TXT)

Dataset S2 Annotation for the Genome-Wide Set of SNPs Ascertained in Laboratory Strains Position is from mouse genome build 36 (mm8). The file contains tab-delimited text in 4,581 rows and three columns.
(104 KB TXT)

Dataset S3 Genotypes for the Genome-Wide Set of SNPs The file contains tab-delimited text in 120 rows and 4,582 columns.
(264 KB ZIP)

Dataset S4 Annotation for the Set of Markers Discovered in Four Regions by Resequencing Arizona Mice The column “local.pos” is the position on a local assembly of sequences, and “mm8.pos” is the position on mouse genome build 36 (mm8). The file contains tab-delimited text in 301 rows and six columns.
(12 KB TXT)

Dataset S5 Genotypes of the Set of Markers Discovered in Arizona Mice by Resequencing The file contains tab-delimited text in 77 rows and 302 columns.
(93 KB TXT)

Figure S1 Genetic Distance versus Collection Site Distance for All 4,371 Pairs of 94 Wild Mice from Arizona (Using 3,928 Autosomal SNPs) (243 KB PDF)

Figure S2 The Results of Analyzing Autosomal Genotypes of Wild Mice Using the Program Structure 2.2 [34] (752 Markers Spaced 2 Mb or More Apart) The result for each independent run of the program (three to seven per model) is indicated by an open black circle, and the mean over runs for a given model is indicated by a solid red circle.
(A) 94 Arizona mice and (B) 120 mice including the 94 Arizona mice and five to seven mice each from the three subspecies from their native ranges (as in Figure 1).
(6 KB PDF)

Figure S3 The Fraction of Loci Homozygous versus the Estimated Inbreeding Coefficient for 94 Mice from Arizona (Using 3,928 Autosomal SNPs) (7 KB PDF)

Figure S4 The Genetic Similarity (Average Fraction of Alleles Shared per Locus) versus the Estimated Kinship Coefficient for All 4,371 Pairs of 94 Arizona Mice (Using 3,928 Autosomal SNPs) The vertical line at 0.76 is the expected genetic similarity of unrelated individuals given the observed allelic frequencies and an inbreeding coefficient of 0.21 (the average of the F estimates for the 94 individuals). The horizontal line is 1/64, the expected kinship coefficient of second cousins in a population without inbreeding; 91% of the kinship coefficient estimates fall below this line.
(241 KB PDF)

Figure S5 LD between Pairs of ∼2,900 Autosomal SNPs in Humans and Mice The *r* ^2^ variable is the composite (genotypic) measure of LD, which is very similar to the usual gametic measure (see Materials and Methods). The jagged red line is the 95th percentile of the *r* ^2^ values within a 25 kb sliding window. The horizontal red line is the genome-wide threshold for significance of *r* ^2^ at α = 0.05 (calculated as the 95th percentile of permuted data). The jagged black line is the mean of the *r* ^2^ values within a 25 kb sliding window. HS is an outbred laboratory strain.
(981 KB PDF)

Figure S6 Comparison of the Composite (Genotypic) *r* ^2^ Measure of LD with the Usual Gametic *r* ^2^
The top panel is data for 2,859 autosomal SNPs in 60 unrelated CEU humans from the International HapMap Project; the bottom panel is data for 2,974 autosomal SNPs in 94 Arizona mice. The straight line has an intercept of 0 and a slope of 1.
(2.3 MB PDF)

### Accession Numbers

The GenBank (http://www.ncbi.nlm.nih.gov/Genbank) accession numbers for the regions resequenced in this study are EU007907 for *Alox15,* EU007908 for *Apoa2,* EU007909 for *C3ar1,* and EU007910 for *Nr1h3.*

## Abbreviations

CEU: Centre d'Etude du Polymorphisme Humain samples of Utah residents with ancestry from northern and western Europe
HS: heterogeneous stock
HW: Hardy-Weinberg
LD: linkage disequilibrium
MAF: minor allele frequency
QTL: quantitative trait loci
SNP: single nucleotide polymorphism
