# Supplementary material for: Linkage Disequilibrium in Wild Mice
Source: PLoS Genet. 2007 Aug 24;3(8):e144. doi: 10.1371/journal.pgen.0030144 (PMC1950958; doi:10.1371/journal.pgen.0030144)
Supplement: Supplementary file 1 [file pgen.0030144.sg001.pdf]

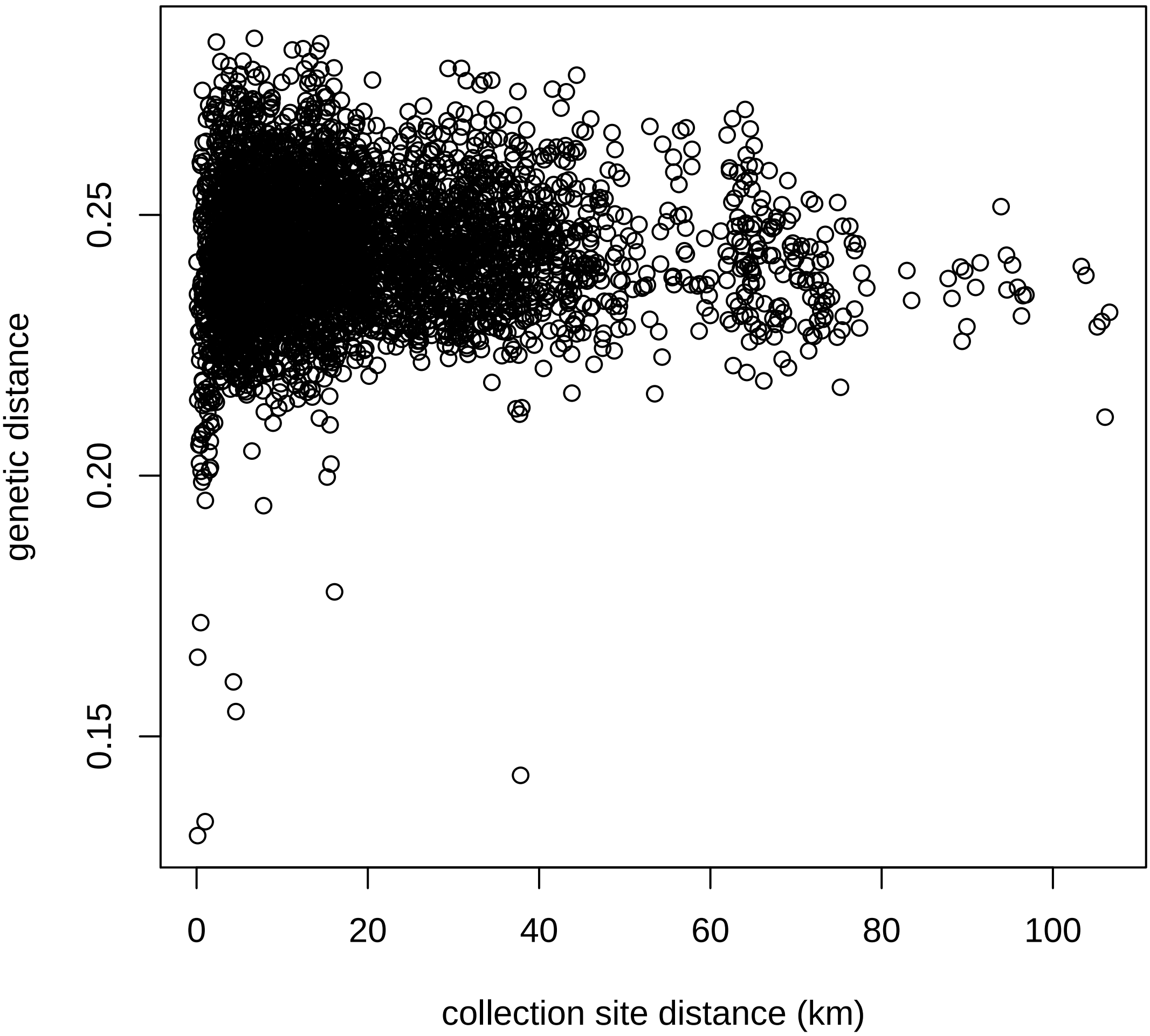

genetic distance
0.15
0.20
0.25
0
20
40
60
80
100
collection site distance (km)
