# Supplementary material for: Linkage Disequilibrium in Wild Mice
Source: PLoS Genet. 2007 Aug 24;3(8):e144. doi: 10.1371/journal.pgen.0030144 (PMC1950958; doi:10.1371/journal.pgen.0030144)
Supplement: Supplementary file 2 [file pgen.0030144.sg002.pdf]

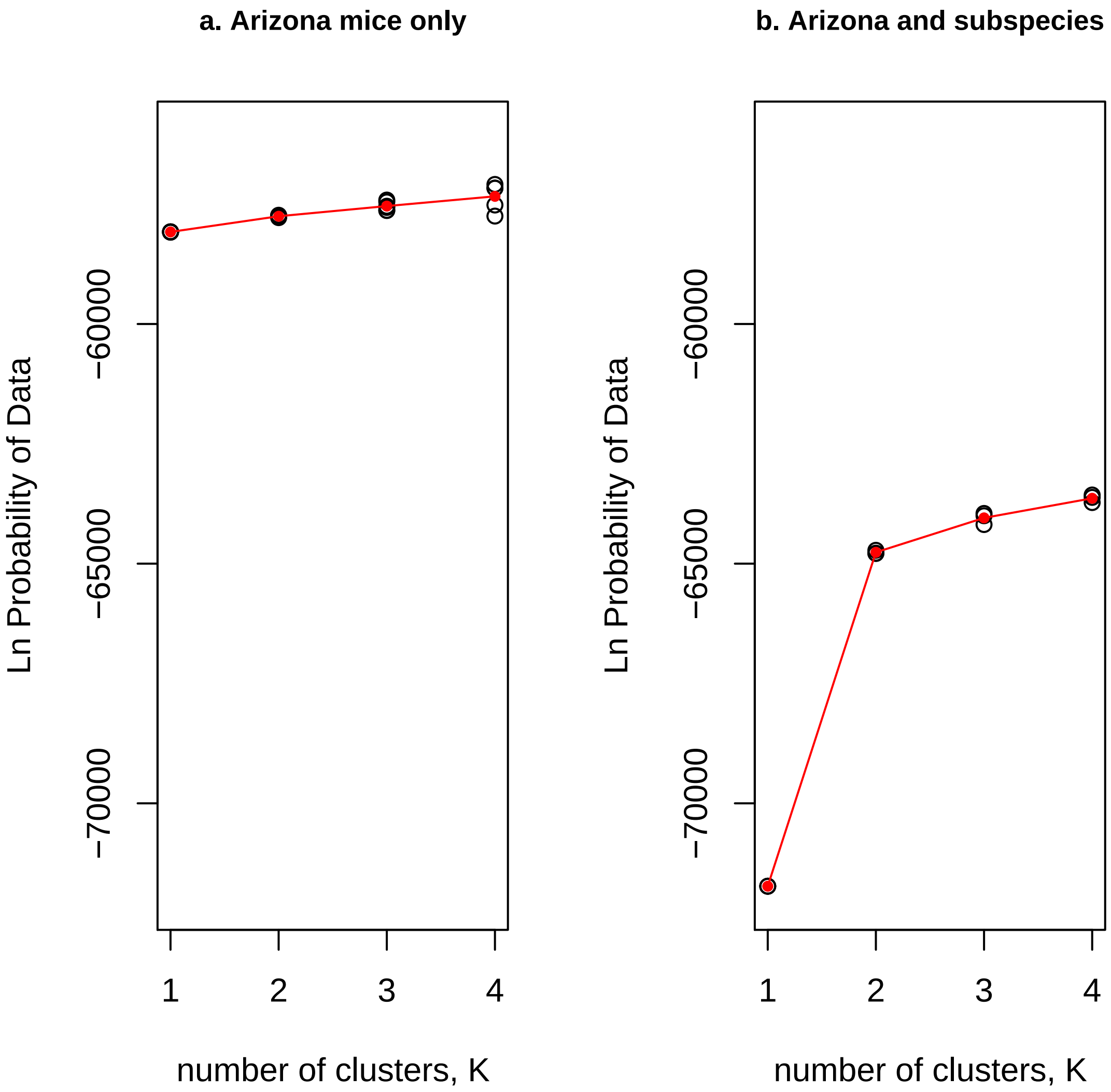
a. Arizona mice only
b. Arizona and subspecies
Ln Probability of Data
−60000
−65000
−70000
1
2
3
4
number of clusters, K
