# Supplementary material for: Linkage Disequilibrium in Wild Mice
Source: PLoS Genet. 2007 Aug 24;3(8):e144. doi: 10.1371/journal.pgen.0030144 (PMC1950958; doi:10.1371/journal.pgen.0030144)
Supplement: Supplementary file 4 [file pgen.0030144.sg004.pdf]

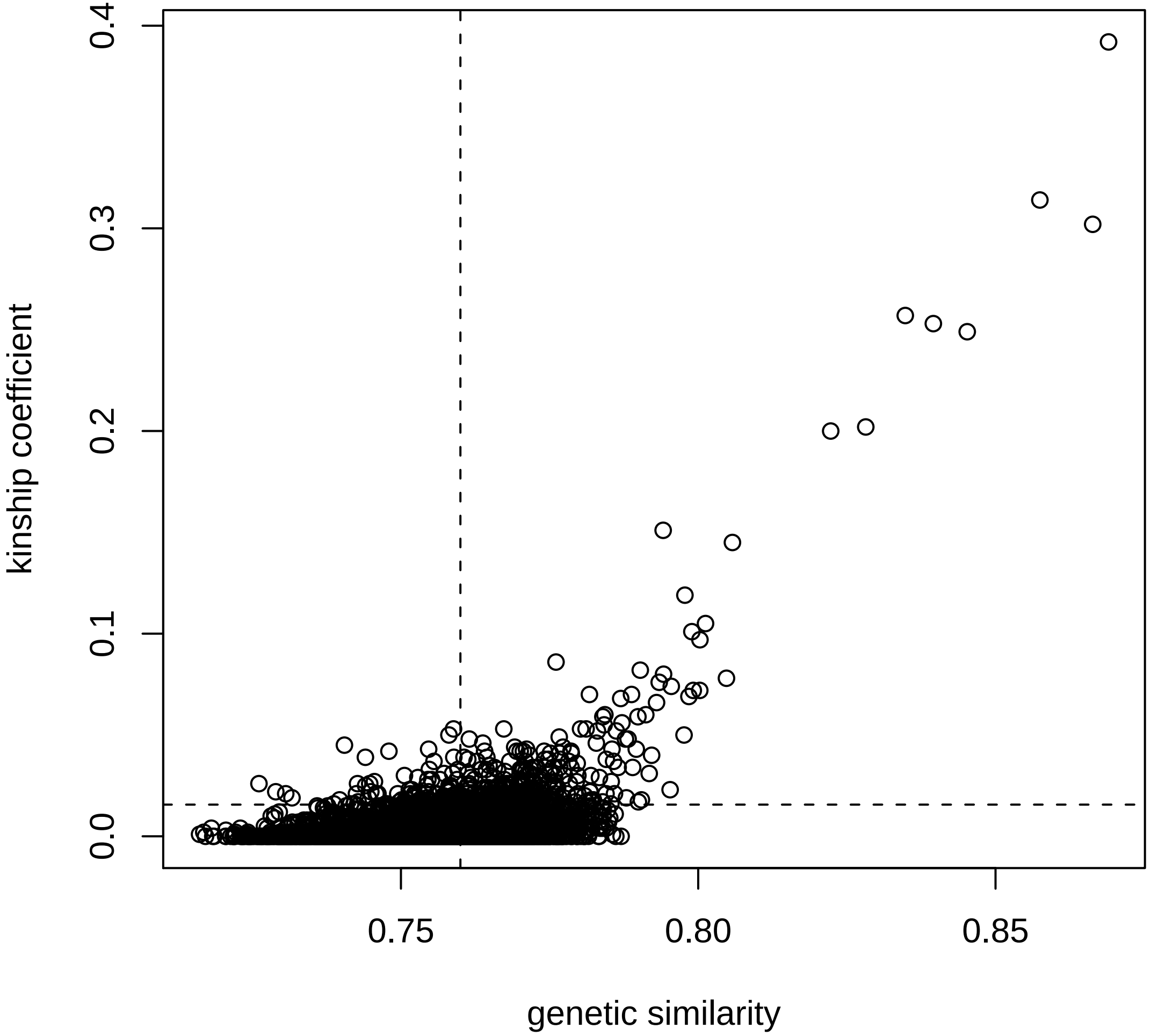

0.0
0.1
0.2
0.3
0.4
kinship coefficient
0.75
0.80
0.85
genetic similarity
