# Supplementary material for: Linkage Disequilibrium in Wild Mice
Source: PLoS Genet. 2007 Aug 24;3(8):e144. doi: 10.1371/journal.pgen.0030144 (PMC1950958; doi:10.1371/journal.pgen.0030144)
Supplement: Supplementary file 5 [file pgen.0030144.sg005.pdf]

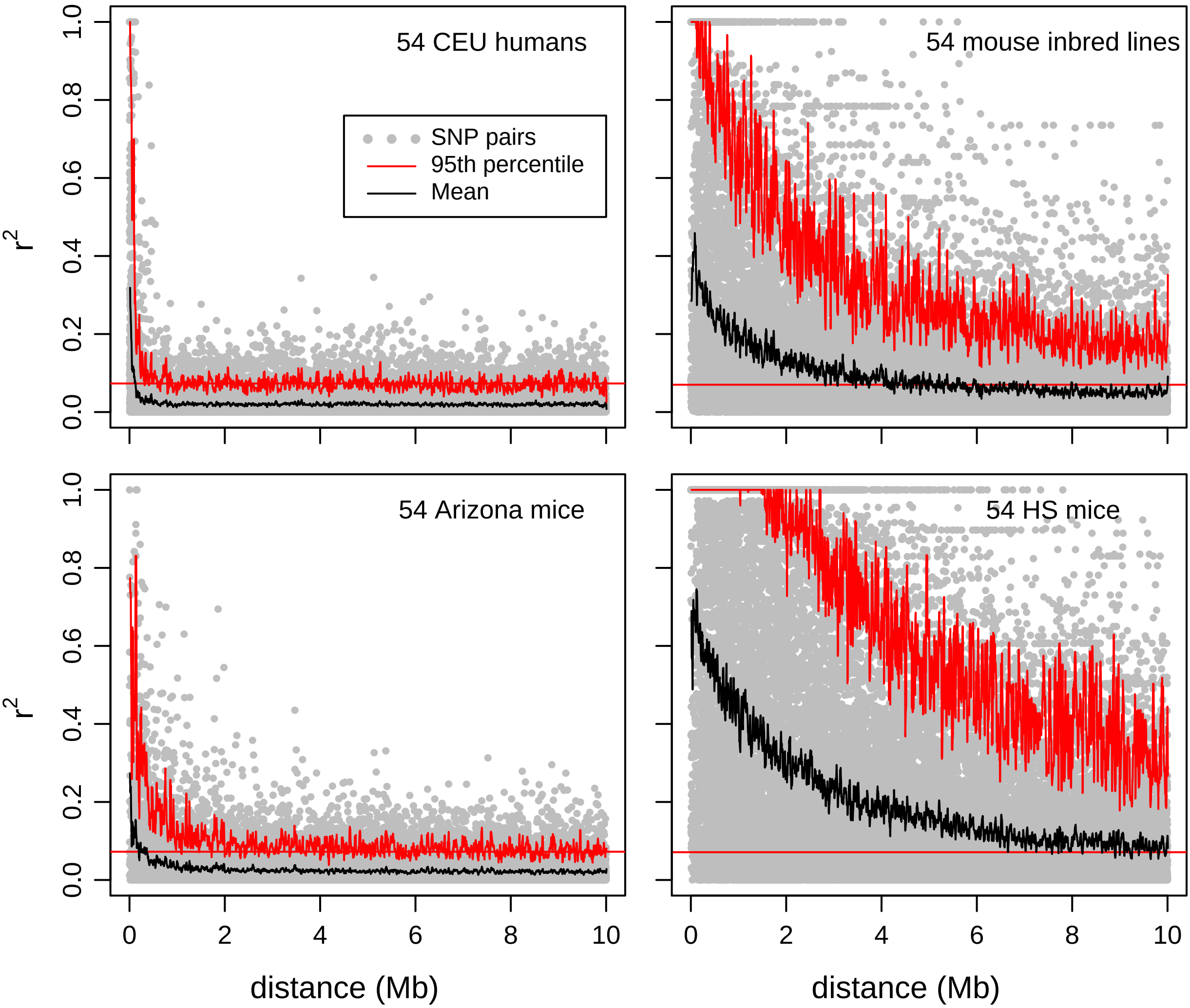

54 CEU humans
SNP pairs
95th percentile
Mean
54 mouse inbred lines
54 Arizona mice
54 HS mice
r2
1.0
0.8
0.6
0.4
0.2
0.0
0
2
4
6
8
10
distance (Mb)
