# Supplementary material for: Linkage Disequilibrium in Wild Mice
Source: PLoS Genet. 2007 Aug 24;3(8):e144. doi: 10.1371/journal.pgen.0030144 (PMC1950958; doi:10.1371/journal.pgen.0030144)
Supplement: Supplementary file 6 [file pgen.0030144.sg006.pdf]

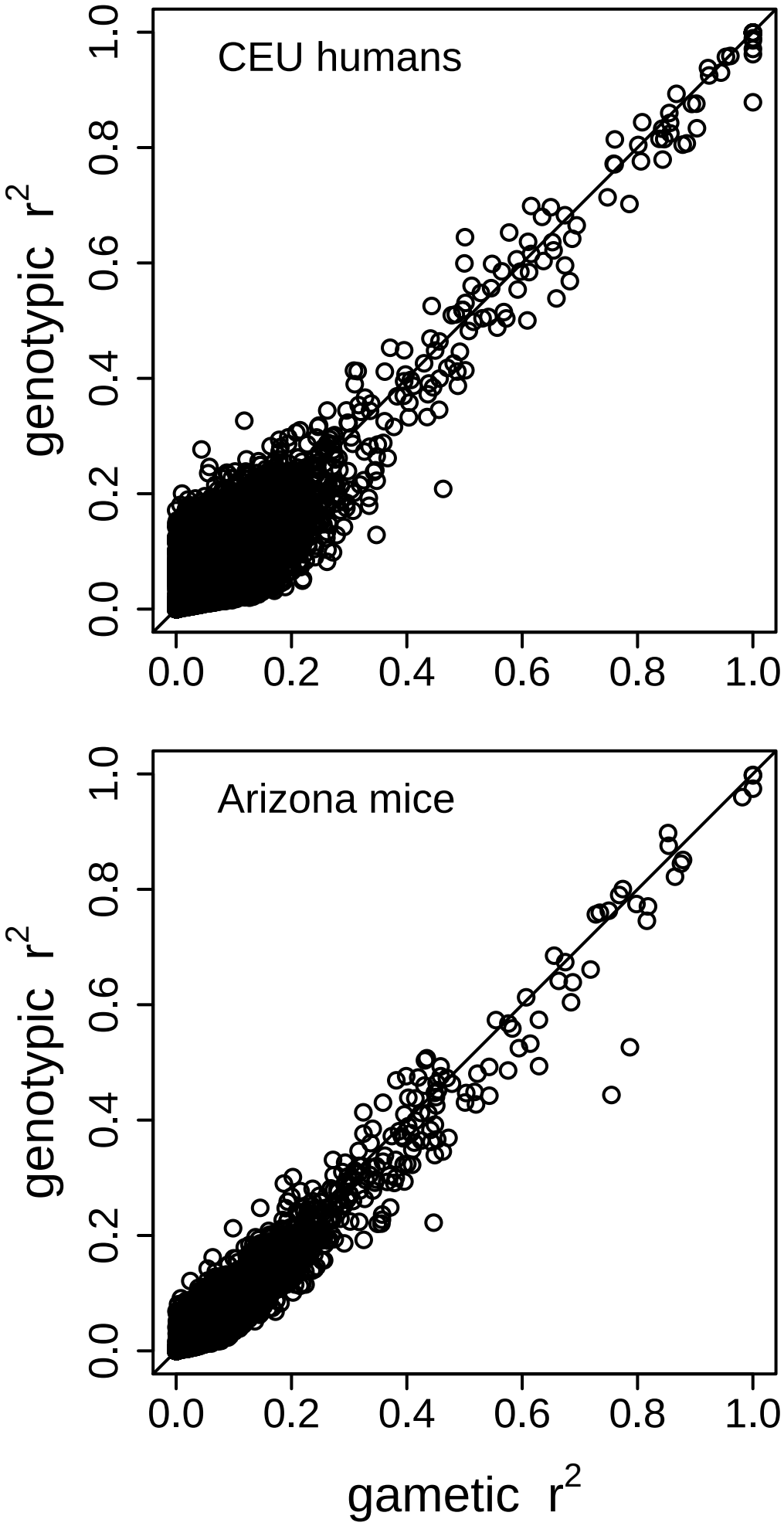

CEU humans
Arizona mice
genotypic r²
gametic r²
0.0
0.2
0.4
0.6
0.8
1.0
